# Combined analysis of circulating epithelial cells and serum thyroglobulin for distinguishing disease status of the patients with papillary thyroid carcinoma

**DOI:** 10.18632/oncotarget.6587

**Published:** 2015-12-13

**Authors:** Hung-Chih Lin, Miaw-Jene Liou, Hsung-Ling Hsu, Jason Chia-Hsun Hsieh, Yi-An Chen, Ching-Ping Tseng, Jen-Der Lin

**Affiliations:** ^1^ Graduate Institute of Biomedical Science, College of Medicine, Chang Gung University, Taoyuan, Taiwan, ROC; ^2^ Division of Endocrinology and Metabolism, Department of Internal Medicine, Chang Gung Memorial Hospital, Taoyuan, Taiwan, ROC; ^3^ Department of Medical Biotechnology and Laboratory Science, College of Medicine, Chang Gung University, Taoyuan, Taiwan, ROC; ^4^ Division of Hematology-Oncology, Department of Internal Medicine, Chang Gung Memorial Hospital, Taoyuan, Taiwan, ROC; ^5^ Department of Chemical and Materials Engineering, Chang Gung University, Taoyuan, Taiwan, ROC; ^6^ Molecular Medicine Research Center, Chang Gung University, Taoyuan, Taiwan, ROC; ^7^ Department of Laboratory Medicine, Chang Gung Memorial Hospital, Taoyuan, Taiwan, ROC

**Keywords:** papillary thyroid carcinoma, thyroid cancer, thyroglobulin, circulating epithelial cells, metastasis

## Abstract

Papillary thyroid carcinoma (PTC) accounts for about 80% of the cases in thyroid cancer. Routine surveillance by serum thyroglobulin (Tg) and medical imaging is the current practice to monitor disease progression of the patients. Whether enumeration of circulating epithelial cells (CECs) helps to define disease status of PTC patients was investigated. CECs were enriched from the peripheral blood of the healthy control subjects (G1, *n* = 17) and the patients at disease-free status (G2, *n* = 26) or with distant metastasis (G3, *n* = 22). The number of CECs expressing epithelial cell adhesion molecule (EpCAM) or thyroid-stimulating hormone receptor (TSHR) was determined by immunofluorescence microscopy analyses. The medium number of EpCAM^+^-CECs was 6 (interquartile range 1-11), 12 (interquartile range 7-16) and 91 (interquartile range 31-206) cells/ml of blood for G1, G2 and G3, respectively. EpCAM^+^-CEC counts were significantly higher in G3 than in G1 (*p* < 0.05) and G2 (*p* < 0.05). The medium number of TSHR^+^-CECs was 9 (interquartile range 3-13), 16 (interquartile range 10-24) and 100 (interquartile range 31-226) cells/ml of blood for G1, G2 and G3, respectively. The TSHR^+^-CEC counts also distinguished G3 from G1 (*p* < 0.05) and G2 (*p* < 0.05). With an appropriate cut off value of CEC count, the disease status for 97.9% (47/48) of the cases was clearly defined. Notably, the metastatic disease for all patients in G3 (22/22) was revealed by combined analysis of serum Tg and CEC. This study implicates that CEC testing can supplement the current standard methods for monitoring disease status of PTC.

## INTRODUCTION

Thyroid cancer is the most common endocrine malignancy worldwide and is the fourth most common cancer in women with a 2.4-fold increase in the annual incidence over the past decade [1–4]. Among the subtypes of thyroid cancer, papillary thyroid carcinoma (PTC) accounts for more than 80% of the cases [5, 6]. The prognosis of PTC is usually favorable. However, recurrence during the first year after initial thyroidectomy is a poor prognostic factor and is a challenge in patient management [7].

A number of PTC patients in remission eventually develop loco-regional recurrence or distant metastasis [8–10]. Routine surveillance of the disease status by serum thyroglobulin (Tg) and medical imaging such as ultrasonography, positron emission tomography (PET), computed tomography (CT), PET-CT, magnetic resonance imaging (MRI), and ^131^I-whole body scintigraphy (^131^I-WBS) is required [11, 12]. However, the presence of anti-Tg antibody (anti-TgAb) in the bloodstream usually interferes with the interpretation of serum Tg testing and the disease status of the patients [13, 14]. Medical imaging studies which are usually performed in an interval of 6-12 months also have limitations in unveiling patient status in real time. The best modality for follow-up of thyroid cancer patients remains to be improved.

Circulating epithelial cells (CECs) or circulating tumor cells are the “liquid biopsies” that serve as a tool to monitor treatment response and disease progression in a number of cancer types [15, 16]. With only few studies addressing the clinical values of CECs in PTC [17], whether CEC testing is able to distinguish disease status of the patients with PTC is still not clear. In this study, we analyzed the number of CECs from 17 healthy control subjects and 48 PTC patients at disease-free status or with distant metastatic disease. We reported that CEC counts were significantly increased in the patients with distant metastasis of which the disease status can be revealed by combined analysis of serum Tg and CECs. The significance of these findings in clinical management of the patients with PTC is discussed.

## RESULTS

### Basic characteristics of enrolled cases

A total of 48 PTC patients and 17 healthy volunteers were enrolled in the study between April 2013 and August 2015 and were categorized into three groups (G1, G2 and G3). G1 included 17 healthy control individuals (three males and fourteen females) who had a median age of 44 (interquartile range 33-53) years (Tables 1 and 2). All healthy control subjects underwent thyroid ultrasonography and blood testing of free thyroxine (T4), thyroid-stimulating hormone (TSH), Tg, and anti-thyroid peroxidase (TPO) antibody (Table 2). Normal levels of serum free T4, TSH and Tg (Table 2), and negative in anti-TPO antibody testing with no obvious nodule in thyroid ultrasonography (data not shown) were demonstrated to exclude any thyroid disorders such as thyroid nodules, autoimmune thyroid disease, and cancer history. G2 included 26 PTC patients (six males and twenty females) who have received curative treatment and were confirmed as disease-free by serum Tg testing and medical imaging studies (Tables 1 and 3). The median age and the median follow-up duration of these patients was 55.5 (interquartile range 45-63) years and 8.7 (interquartile range 5.4-12.5) years, respectively. The PTC subtypes of these patients included classical PTC (*n* = 20), follicular variant of PTC (FVPTC, *n* = 2), multifocal classical PTC (*n* = 2) and multifocal FVPTC (*n* = 2). All other clinical features including TNM staging, the surgery received by the patients, the levels of anti-TgAb, the follow-up duration, and the cumulative radioactive iodine (RAI) dose received by the patients were described in Table 3. G3 included 22 patients (six males and sixteen females) who were confirmed to have distant metastatic disease (Tables 1 and 4). Distant metastases were demonstrated by histopathology using immunochemical staining (cases #1 and #13 of G3), ^131^I-WBS or other imaging methods including CT, PET-CT, MRI, X-ray, and bone scan. The median age and the median follow-up duration of these patients was 52 (interquartile range 38-75) years and 6.6 (interquartile range 1.3-11.0) years, respectively. The PTC subtypes of these patients included classical PTC (*n* = 8), FVPTC (*n* = 4), multifocal classical PTC (*n* = 7), multifocal FVPTC (*n* = 1), multifocal vascular PTC (*n* = 1) and poorly differentiated thyroid carcinoma (PDTC, *n* = 1). All other clinical features including TNM staging, the imaging methods to confirm the status of distant metastasis, the surgery received by the patients, the sites of metastasis, the levels of serum Tg and anti-TgAb, the follow-up duration, and the cumulative RAI dose received by the patients were described in Table 4. The enrolled cases were age- and gender-matched among the three groups with no statistical difference for the follow-up duration between G2 and G3 (Table 1).

**Table 1:** Basic characteristics of the study subjects

Parameter	G1	G2	G3	*p*-value
Age (year)^a^	44 (33-53)	55.5 (45-63)	52 (38-75)	0.0582
Gender (male/female)	3/14	6/20	6/16	0.7816
Follow-up duration (year)^a^	-	8.7 (5.4-12.5)	6.6 (1.3-11.0)	0.1923
Cumulative RAI dose (mCi)^a^	-	60 (30-60)	195 (90-650)	0.0001

a The data represent the median and the interquartile range for the indicated parameters.

**Table 2:** Clinical features, serum markers and CEC counts for the control subjects in G1

No.	Gender	Age (year)	Free T4 (ng/dl)	TSH (μIU/ml)	Tg (ng/ml)	EpCAM^+^-CECs (cell/ml)	TSHR^+^-CECs (cell/ml)
1	F	50	ND^a^	1.112	11.83	6	6
2	F	52	ND	2.018	13.34	2	5
3	F	44	ND	1.506	14.81	16	12
4	F	72	ND	0.889	74.76	0	2
5	F	33	ND	1.729	9.45	1	1
6	F	42	1.13	0.733	0.71	11	20
7	F	34	ND	1.593	7.22	1	1
8	F	33	1.11	0.852	5.88	1	7
9	F	25	ND	1.918	28.42	4	12
10	F	23	1.55	1.463	4.48	14	19
11	F	52	1.15	1.318	5.40	14	21
12	F	54	1.18	2.155	7.24	11	13
13	F	54	1.32	2.157	16.86	10	12
14	F	51	1.15	2.471	5.13	11	9
15	M	42	1.49	1.374	9.51	9	2
16	M	56	1.17	1.523	1.83	1	7
17	M	30	1.19	1.235	7.18	2	14

a Not determined.

**Table 3:** Clinical features, treatment and CEC counts for the PTC patients in G2

No.	Gender	Age (year)	Histological variant	TNM stage	Surgery	Anti-TgAb (IU/ml)	Follow-up (year)	Cumulative RAI dose (mCi)	EpCAM^+^ (cell/ml)	TSHR^+^ (cell/ml)
1	F	52	Classical	T2N0M0	TT	< 10.00	2.3	60	2	17
2	F	36	Classical	T1N0M0	TT	< 10.00	9.1	60	5	10
3	F	48	Classical	T1N0M0	TT	< 10.00	11.6	30	10	15
4	F	44	Classical	T1N1M0	TT with LND	19.43	7.6	160	4	4
5	F	63	Classical	T1N0M0	ST	13.70	16.8	60	14	13
6	F	45	Classical	T1N0M0	TT	< 10.00	2.4	60	49	11
7	F	56	Classical	T1N0M0	TT	20.34	10.5	30	13	18
8	F	72	Classical	T1N0M0	TT	< 10.00	11.8	60	9	10
9	F	74	Classical	T2N0M0	TT	22.24	15.2	30	7	8
10	F	71	Classical	T1N0M0	ST	< 10.00	15.1	60	17	22
11	F	43	Classical	T1N0M0	ST	10.57	10.1	30	19	32
12	F	60	Classical	T1N0M0	TT	12.13	12.9	30	22	29
13	F	55	Classical	T1N0M0	TT	12.24	6.8	30	9	14
14	F	35	Classical	T3N0M0	TT	< 10.00	12.6	60	9	11
15	F	41	Classical	T1N0M0	TT	13.45	6.6	30	22	29
16	M	59	Classical	T3N0M0	TT with LND	< 10.00	5.5	60	5	1
17	M	48	Classical	T1N0M0	TT	13.28	2.7	60	14	17
18	M	63	Classical	T2N0M0	TT	11.27	16.0	90	16	18
19	M	58	Classical	T2N0M0	TT	21.36	6.8	30	16	19
20	M	60	Classical	T1N0M0	TT	11.99	12.4	60	8	8
21	F	55	FVPTC	T3N0M0	TT	12.30	3.7	60	14	38
22	F	57	FVPTC	T2N0M0	TT	13.46	9.5	30	15	24
23	F	64	Classical, multifocal	T1N0M0	TT	17.13	6.2	30	11	10
24	M	66	Classical, multifocal	T1N0M0	TT	13.21	5.0	60	7	4
25	F	53	FVPTC, multifocal	T1N0M0	TT	14.49	3.9	60	26	30
26	F	37	FVPTC, multifocal	T2N0M0	TT	17.67	8.2	30	2	24

Abbreviations: TT, total thyroidectomy; ST, subtotal thyroidectomy; LND, lymph-node dissection.

**Table 4:** Clinical features, treatment and CEC counts for the PTC patients in G3

No.	Gender	Age (year)	Histological variant	TNM stage	Imaging		Surgery	Site of metastasis	Tg (ng/ml)	Anti-TgAb (IU/ml)	Follow-up (year)	Cumulative RAI dose (mCi	EpCAM^+^ (cell/ml)	TSHR^+^ (cell/ml)
					^131^I-WBS	Others								
1	F	49	Classical	T2N0M0	-	+ (CT)	TT with LND	L	0.11	22.19	13.3	60	953	351
2	F	30	Classical	T2N1bM0	+	+ (PET-CT)	TT with LND	L, M	16.3	<10.00	5.7	660	41	92
3	F	64	Classical	T3N1M0	+	+ (CT)	TT with LND	L	179	21.01	11.3	970	211	22
4	F	30	Classical	T2N1M0	+	- (PET-CT)	TT with LND	L	3.33	15.25	9.7	190	30	30
5	F	97	Classical	T2N0M0	+	+ (CT)	ST	L, B	624.83	21.54	14.3	220	31	13
6	M	15	Classical	T4N1M1	+	- (CT)	TT with LND	L	102	24.27	1	100	243	106
7	M	67	Classical	T3N0M1	-	+ (CT, MRI, X ray)	TT with LND	L	69.38	14.35	0.3	0	208	336
8	M	22	Classical	T4N0M0	+	+ (CT, PET-CT)	TT with LND	L	0.39	17.09	10.9	630	205	75
9	F	41	FVPTC	T3N0M1	+	+ (Bone scan)	TT	B	0.56	12.99	7.4	650	79	239
10	F	68	FVPTC	T3N0M1	+	+ (CT)	TT	B, L	Undetectable	919.1	1.4	130	100	152
11	F	75	FVPTC	TxNxM1	+	+ (CT)	TT	K, L	Undetectable	20.59	6.4	120	197	223
12	M	51	FVPTC	T4N0M0	+	+ (X-ray)	TT with LND	L	5.38	28.4	5	190	22	2
13	F	87	Classical, multifocal	T4N1M0	-	+ (CT, X ray)	TT	L	95.5	14.85	10.8	210	53	65
14	F	79	Classical, multifocal	T4N0M0	+	+ (CT, MRI)	TT	B, M	6258	67.21	16.1	300	82	94
15	F	74	Classical, multifocal	T4N1M0	+	+ (CT)	TT	L, M	10.5	13.7	6.8	680	226	235
16	F	41	Classical, multifocal	T1N1M0	+	+ (CT, PET-CT)	TT with LND	L, M	20.3	16.03	8.7	910	11	32
17	F	49	Classical, multifocal	T4N1M0	+	- (CT)	TT with LND	L	59.31	18.39	14.4	655	7	5
18	F	79	Classical, multifocal	T3N0M0	-	+ (CT)	TT with LND	L, M	60.62	22.16	0.5	30	113	860
19	M	51	Classical, multifocal	T2N0M1	+	+ (CT, MRI, Bone scan)	TT	L, B	14609	138.4	0.5	0	167	113
20	M	53	FVPTC, multifocal	T4N1M0	+	+ (CT)	TT with LND	Brain	127	14.31	3.1	100	74	136
21	F	16	Vascular, multifocal	T4N1M1	+	+ (CT)	TT with LND	L	8.21	15.94	1.1	200	23	34
22	F	75	PDTC	T4NxM1	-	+ (CT)	-	L	11346	18.34	6.3	30	190	162

Abbreviations: ST, subtotal thyroidectomy; TT, total thyroidectomy; LND, lymph-node dissection; B, bone; K, kidney; L, lung; M, mediastinum.

### PTC patients with distant metastasis had an increase in EpCAM^+^-CEC and TSHR^+^-CEC counts

**Figure 1:**
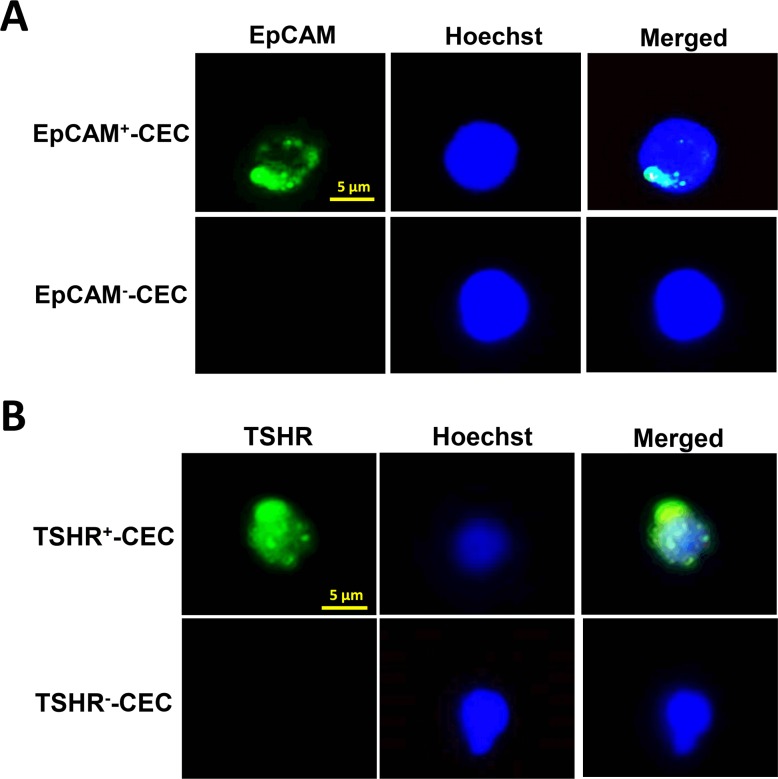
Isolation and characterization of CECs from patients with PTC **A.**-**B.** CECs were isolated by PowerMag system and analyzed by immunofluorescence staining as described in the Materials and Methods. At least two CEC populations that were positive for EpCAM (panel A, green) or positive for TSHR (panel B, green), were defined. Positive staining of Hoechst 33342 (panel A and B, blue) indicates the presence of intact nucleated cells.

After RBC lysis and removal of CD45^+^ white blood cells from the peripheral blood by the PowerMag system, the enriched cells were analyzed by immunofluorescence staining using the antibody against epithelial cell adhesion molecule (EpCAM) and thyroid-stimulating hormone receptor (TSHR), the marker for the cells originated from epithelia and thyroid, respectively [18, 19]. Staining of the cells with Hochest 33342 DNA staining dye was used to define the nucleated cells. Fluorescence microscopy analysis was performed to identify the CECs that expressed EpCAM or TSHR. Representative CECs that were positive for EpCAM or TSHR were shown (Figure 1).

**Figure 2:**
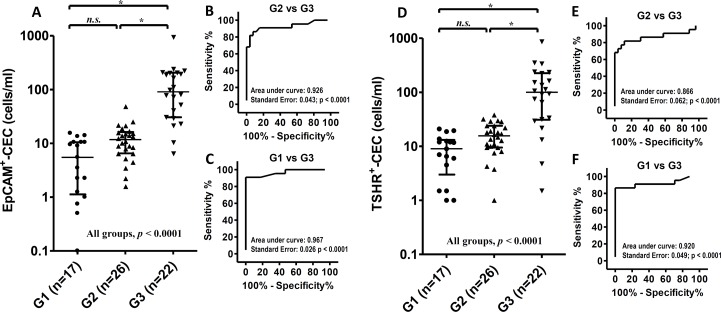
Scatter dot plots and ROC analyses for EpCAM^+^-CEC and TSHR^+^-CEC counts in the healthy controls and the patients with PTC **A.**-**F.** The scatter dot plots for the number of EpCAM^+^-CEC (panel A) and TSHR^+^-CEC (panel D) in G1, G2 and G3. Kruskal-Wallis test with the post-hoc Dunn's test were used for statistical analyses. The medium and the interquartile range for each group are indicated by the horizontal lines. ROC analysis for the number of EpCAM^+^-CEC (panel B and C) and TSHR^+^-CEC (panel E and F) between G2 and G3 (panel B and E), and between G1 and G3 (panel C and F).

The number of CECs per ml of blood was determined and compared among the healthy controls (G1), the PTC patients at disease-free status (G2) and the patients with distant metastasis (G3). The median number of EpCAM^+^-CECs was 6 (interquartile range 1-11), 12 (interquartile range 7-16) and 91 (interquartile range 31-206) cells/ml for G1, G2 and G3, respectively (Figure 2A). EpCAM^+^-CEC count was significant difference between G2 and G3 (*p* < 0.05), between G1 and G3 (*p* < 0.05), and among G1, G2 and G3 (*p* < 0.0001). However, it was not significant difference between G1 and G2. Receiver operating characteristic (ROC) analysis revealed that EpCAM^+^-CEC count distinguished G3 from G2 with an area under the curve (AUC) equivalent of 0.926 (*p* < 0.0001) (Figure 2B). The sensitivity and specificity of the assay was 86.4% and 92.3%, respectively, when the cut off value was 22 EpCAM^+^-CECs/ml (Table 5 and Supplementary Table 1). On the other hand, EpCAM^+^-CEC count distinguished G3 from G1 with the AUC equivalent of 0.967 (*p* < 0.0001) (Figure 2C). The sensitivity and specificity of the assay was 90.9% and 100%, respectively, when the cut off value was 16 EpCAM^+^-CECs/ml (Supplementary Table 2).

**Table 5:** ROC analyses of the indicated testing methods in distinguishing G3 from G2

Testing methods^a^	Sensitivity (%)	Specificity (%)	AUC	*p*-value
Medical imaging	100.0	100.0	1.000	< 0.0001
Tg	90.9	100.0	0.955	< 0.0001
EpCAM^+^-CEC	86.4	92.3	0.926	< 0.0001
TSHR^+^-CEC	72.7	96.2	0.866	< 0.0001
Tg/EpCAM^+^-CEC	100.0	92.3	0.962	< 0.0001
Tg/TSHR^+^-CEC	100.0	96.2	0.981	< 0.0001

a CEC testing was considered positive when EpCAM^+^-CEC >22 cells/ml or TSHR^+^-CEC >33 cells/ml.

The number of CECs that expressed TSHR was also analyzed and compared among G1, G2 and G3. The median number of TSHR^+^-CECs was 9 (interquartile range 3-13), 16 (interquartile range 10-24) and 100 (interquartile range 31-226) cells/ml for G1, G2 and G3, respectively (Figure 2D). The TSHR^+^-CEC count was significant difference between G2 and G3 (*p* <0.05), between G1 and G3 (*p* < 0.05), and among G1, G2 and G3 (*p* < 0.0001). However, it was not significant difference between G1 and G2. ROC analysis showed that TSHR^+^-CEC count distinguished G3 from G2 with the AUC equivalent of 0.866 (*p* < 0.0001) (Figure 2E). The sensitivity and specificity of the assay was 72.7% and 96.2%, respectively, when the cut off value was 33 TSHR^+^-CECs/ml (Table 5 and Supplementary Table 3). On the other hand, the TSHR^+^-CEC count distinguished G3 from G1 with the AUC equivalent of 0.920 (*p* < 0.0001) (Figure 2F). The sensitivity and the specificity of the assay was 86.4% and 100%, respectively, when the cut off value was 21 TSHR^+^-CECs/ml (Supplementary Table 4). These data together indicate that the number of EpCAM^+^-CECs and TSHR^+^-CECs is comparable between healthy controls and the patients at disease-free status, but is significantly increased in the PTC patients with distant metastasis.

### CEC counts revealed distant metastatic status of the patients who had undetectable serum Tg

Serum Tg is mainly used for monitoring disease status of post-thyroidectomy patients. In this study, serum Tg was measured when the patients underwent T4 treatment to facilitate the comparison among G1, G2 and G3. Under this circumstance, all patients at disease-free status (G2) had undetectable serum Tg (< 0.1 ng/ml, data not shown). The levels of anti-Tg Ab for all patients in G2 were below 115 IU/ml (Table 3) which was considered in our hospital as the cut off value for no interference with the measurement of serum Tg. This was in accord with the report that the level of anti-TgAb below 30 IU/ml did not interfere with serum Tg testing [13]. Only minimal interference with serum Tg was observed even when the anti-TgAb reached 100 IU/ml [13]. The undetectable serum Tg (< 0.1 ng/ml) for the patients in G2 thereby was not due to the interference by anti-TgAb. On the other hand, 20 of the 22 (90.9%) patients with distant metastasis (G3) had detectable serum Tg (> 0.1 ng/ml) (Table 4). ROC analysis revealed that serum Tg distinguished the patients with distant metastatic disease from the patients at disease-free status with the AUC equivalent of 0.955 (*p* < 0.0001). The sensitivity and specificity of the assay was 90.9% and 100%, respectively (Table 5).

**Figure 3:**
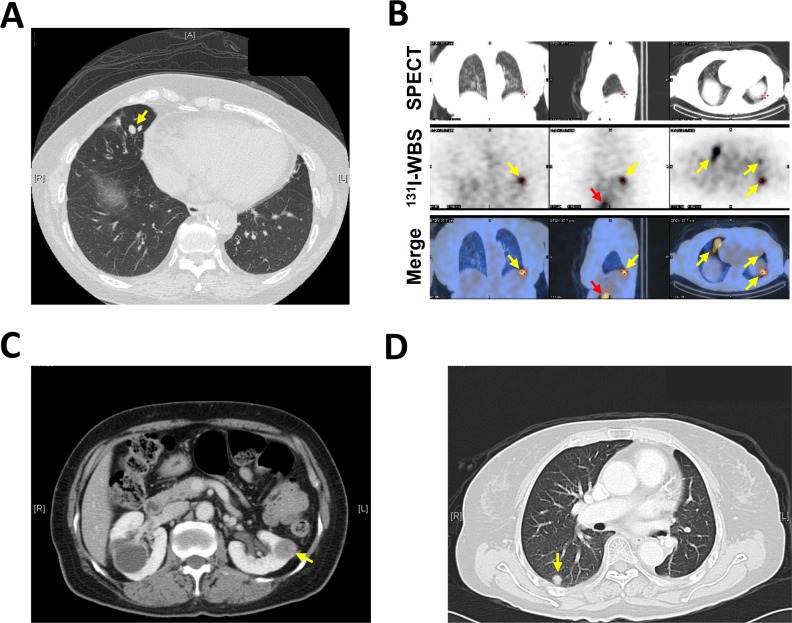
Key medical images reveal disease recurrence in cases #10 and #11 **A.** The image of chest CT demonstrated multiple, round but small (all less than 1 cm in diameter) pulmonary nodules (the yellow arrow indicates one of them) of case #10. These nodules were not distinguishable from the nodules derived from benign diseases such as tuberculosis. **B.** Single-photon emission computed tomography (SPECT, upper row) and ^131^I-WBS (middle low) were performed for case #10 at the 7^th^ day after an oral dose of 100 mCi ^131^I. The images were superimposed (bottom row) to show the multiple focal areas of increased uptake in lung (yellow arrows) and right pelvis (red arrows). The coronal view (left panel), sagittal view (middle panel) and transverse view (right panel) of the images are shown. **C.** The abdominal CT image for case #11 shows a left renal middle pole tumor (2.2 × 1.7 cm) which was undistinguishable from a primary renal cell carcinoma. **D.** The chest CT image for case #11 shows one of the multiple small and round opacities in the lung (yellow arrow).

The cases #10 and #11 in G3 were the two patients with undetectable Tg. Case #10 was a 68 year-old female diagnosed with PTC without capsular or stromal invasion. After thyroidectomy, no evidence of loco-regional and distant metastasis was revealed by ^131^I-WBS and neck CT. The patient was enrolled in the study one year after thyroidectomy. Serum Tg of the patient was undetectable (< 0.1 ng/ml) but anti-TgAb was positive (919.1 IU/ml). However, CEC count was significantly elevated in this patient. The number of EpCAM^+^-CECs and TSHR^+^-CECs was 100 cells/ml and 152 cells/ml, respectively (Table 4). Based on these findings, CT scan (Figure 3A) and ^131^I-WBS with the therapeutic dose of 100 mCi (Figure 3B) were performed that ultimately confirmed the patient had multiple lung and right pelvis bone metastases.

For the case #11, the anti-TgAb was low (20.59 IU/ml) that did not contribute to the undetectable serum Tg for the patient. In accord with this notion, serum Tg was detectable (9.06 ng/ml) in another blood test when T4 treatment was interrupted. The patient had significantly elevated CEC counts with the number of EpCAM^+^-CECs and TSHR^+^-CECs equivalent of 197 cells/ml and 223 cells/ml, respectively (Table 4). The patient was subsequently revealed to have kidney and lung metastases by ^131^I-WBS and CT (Figure 3C and 3D). Histological proof of FVPTC was confirmed after partial left nephrectomy (image not shown). The data of these two patients with undetectable serum Tg together indicate that CEC enumeration could supplement serum Tg testing and standard imaging methods in defining metastatic status of the patients.

### Comparison of CEC testing with conventional methods in distinguishing disease status of the patients with PTC

Imaging studies such as CT/MRI/PET and ^131^I-WBS are the gold standard for detection of recurrent disease when serum Tg is detectable [11]. Although the sensitivity and specificity for the combination of standard imaging methods in the identification of distant metastasis were both 100% (Table 5), the risk in radiation exposure and the costs associated with the examinations lead us to determine whether combined analysis of serum Tg with CECs is an alternative way to monitor disease status of the patients with PTC. Our data revealed that combined analysis of serum Tg with EpCAM^+^-CEC or TSHR^+^-CEC defined the disease status (disease-free vs. distant metastasis) in 46 (95.8%) or 47 (97.9%) of the 48 patients, respectively (Tables 3 and 4). Notably, metastatic status of all 22 patients (100%) in G3 was revealed by combined analyses of serum Tg and CECs. ROC analyses showed that combined analysis of serum Tg with EpCAM^+^-CEC distinguished distant metastasis from disease-free status with the AUC equivalent of 0.962 (*p* < 0.0001). The sensitivity and specificity of the assay was 100% and 92.3%, respectively. When combined analysis of serum Tg with TSHR^+^-CEC, the AUC, sensitivity and specificity was 0.981 (*p* < 0.0001), 100% and 96.2%, respectively (Table 5). These data demonstrate that combined analysis of serum Tg with CECs is suitable to distinguish the patients at disease-free status from the patients with distant metastasis.

## DISCUSSION

The prevalence of PTC was increased in many developed and developing countries in recent decades. Although most of the patients with PTC have excellent prognosis after appropriate therapy, some of the patients have poor prognosis [20, 21] and eventually develop local regional recurrence or distant metastases [22, 23]. In this study, we show that CEC enumeration has the potential to compromise the current methods for following up PTC patients at risk of recurrence and distant metastasis.

Immunofluorescence staining of thyroid-related proteins allows us to define the thyroid origin of CECs. TSHR, a general marker for the cells with thyroid origin [19], was used to define circulating thyroid cells in the patients with PTC. Although TSHR is also present in the cells of non-thyroid origin [24], TSHR^+^-CECs were detectable in all metastatic PTC patients [25]. The expression of TSHR mRNA in the circulating cells has also been shown to enhance preoperative detection of cancer in patients with thyroid nodules [26]. We found in this study that TSHR^+^-CECs instead of EpCAM^+^-CECs are more frequently present in most of the PTC patients, implying that not all TSHR^+^-CECs are EpCAM-positive. In support of this notion, co-immunofluorescence staining of CECs using anti-TSHR and anti-EpCAM antibodies reveals a subgroup of CECs that are TSHR-positive but EpCAM-negative (data not shown). This subgroup of CECs is not likely isolated and identified by the CEC enrichment methods based on positive selection of EpCAM^+^ cells [15, 27]. The negative selection system PowerMag we used in this study thereby offers benefits for isolating different populations of CECs including both EpCAM^+^-CECs and EpCAM--CECs.

CEC counts allow real-time monitoring of cancer progression for various cancer types [15, 16, 28]. In this study, we found that the number of EpCAM^+^-CECs and TSHR^+^-CECs is statistically higher for the patients with distant metastasis (G3) than the patients in disease-free status (G2) and the healthy controls (G1). Although it is not statistical significance, the number of TSHR^+^-CECs and EpCAM^+^-CECs is higher for the patients in G2 than in G1. These observations indicate that some of the patients in G2 may carry circulating thyroid cancer cells in the bloodstream, despite that they are negative in histopathology, serum Tg, and ^131^I-WBS. These patients are likely at risk of recurrence and require medical attention. The clearest example is the case #20 in G3. When enrolled in this study, case #20 is presumably disease-free based on the patient's historical data of medical examinations. An unexpected high CEC count (EpCAM^+^-CECs: 74 cells/ml; TSHR^+^-CECs: 136 cells/ml) in this case led to a serial of examinations that ultimately confirmed the diagnosis of PTC with brain metastasis. Similar to the findings in other cancer types [29, 30], CEC testing is an important tool for dynamic monitoring of thyroid cancer progression.

To further confirm the clinical value of CEC enumeration in monitoring the progression of PTC patients, CEC testing was first compared with the standard imaging methods. Because standard imaging methods are the gold standard for monitoring metastatic disease in current clinical practice [11], the AUC, sensitivity and specificity, as expected, was 1.000, 100% and 100% respectively. In contrast, the assay specificity of CEC testing is slightly less than the standard imaging methods (92.3% for EpCAM^+^-CEC and 96.2% for TSHR^+^-CEC vs. 100% for imaging methods). The discrepancy was due to the high EpCAM^+^-CEC and TSHR^+^-CEC count in two and one of the patients in G2, respectively. Whether the cases were at risk of recurrence or were false positive in CEC testing is not clear. Nevertheless, we have noticed that the percentage of disease-free patients with positive CEC testing results (G2, 4.5%) was within the range of the reported recurrence rate (1.4-14%) for the patients with initial remission after surgery and RAI therapy [8–10]. Accordingly, longitudinal follow-up of the patients in G2 who have high CEC count may be required to determine whether CEC testing is valuable to supplement standard imaging methods in the follow-up of patients with no defined metastatic lesion.

Serum Tg is a routine test for monitoring disease progression in PTC patients with total thyroidectomy [11]. The clinical value of CEC testing was then compared with serum Tg assay in this study. Notably, we demonstrated that CEC testing revealed the distant metastatic status for the two patients in G3 (cases #10 and 11) whose serum Tg was undetectable. The anti-TgAb (> 115 IU/ml) which is present in approximately 15% of thyroid cancer patients [31] has been shown to interfere with the measurement of serum Tg leading to false negative Tg testing [32]. The poorly differentiate thyroid cells may also associate with an undetectable level of serum Tg [33]. The association of the high CEC count with the distant metastatic disease of PTC patients indicates that CEC testing is useful in monitoring disease status when the individual has undetectable serum Tg.

The potential application for combined analyses of CECs and serum Tg in distinguishing disease status of the patients with PTC was also evaluated. For the known cases of thyroid cancer patients, sensitivity is relatively more important than specificity in monitoring of disease progression. Our data indicate that all metastatic cases shown by serum Tg and standard imaging methods can be revealed by combined serum Tg and CEC testing. The assay sensitivity for combined analysis of CEC and serum Tg (100%) is comparable to the standard imaging methods (100%) and is superior to serum Tg alone (90.9%). Despite future trials are still required, combined analyses of CEC and serum Tg have the potential to real-time follow up of the patients with PTC and reduce the use of cumbersome and expensive imaging methods.

In conclusion, with the strong correlation between CEC count and the disease status of PTC patients, CEC enumeration is a simple test that can be performed more frequently than standard imaging methods in distinguishing disease status of PTC. Simultaneous measurements of serum Tg and CEC with one blood draw have the potential in follow-up of thyroid cancer progression and management of patient care.

## MATERIALS AND METHODS

### Study subjects

This study was approved by the Chang Gung Memorial Hospital Institutional Review Board (approval ID: 102-3433B). Patients enrollment criteria included (1) age ≧ 18 years and (2) ability of the patients to understand and sign the informed consent. Enrolled cases were classified into three groups. Group I (G1) included control subjects without clinically significant thyroid disorders such as thyroid nodules, autoimmune thyroid disease and cancer history. Thyroid ultrasonography was performed and the serum TSH, Tg, and anti-TPO antibody were measured to confirm the absence of thyroid diseases. Group II (G2) included PTC patients in disease-free status that have been followed up for more than 2 years. Disease-free was defined as serum Tg < 0.1 ng/ml, anti-TgAb < 115 IU/ml, negative in neck ultrasonic examination, negative in chest x-ray examination and negative in ^131^I-WBS. Group III (G3) included PTC patients with distant metastasis that was defined by serum Tg > 0.1 ng/ml when anti-TgAb < 115 IU/ml and positive medical images in ^131^I-WBS, CT/MRI/PET-CT, chest radiography, or bone scan. The patients with cumulative RAI dose more than 1000 mCi were excluded from the study to rule out any potential effect of high RAI dose on CEC enumeration [34]. Neck ultrasonography studies were conducted on a real-time ultrasonographic machine and a 10 MHz transducer (Aloka).

### Thyroid-related biochemical testing

Anti-TgAb was measured by using a competitive radioimmunoassay (Biocode Hycel, Liege, Belgium). The analytical sensitivity is 6 IU/ml. In our hospital, anti-TgAb <115 IU/ml was considered as no interference with serum Tg testing. Anti-TPO antibody was measured by using the anti-TPO Test Kit (Thermo Fisher Scientific, Pittsburgh, PA). Serum TSH and free T4 were measured by using the Siemens Thyroid Assay Reagents (Siemens, Erlangen, Germany). Serum Tg was measured by using the highly sensitive Tg Access assay (Beckman Coulter, Brea, CA). To avoid interference from elevated TSH and facilitate the comparison among G1, G2 and G3, serum Tg was measured when the patients underwent T4 treatment. The blood samples for CEC testing, serum Tg, and other thyroid-related assays were collected simultaneously from one blood drawing.

### Treatment of papillary thyroid cancer patients

The staging of PTC patients was determined using the International Union Against Cancer Tumor-Node-Metastasis (TNM) criteria (6^th^ edition) [35]. All thyroid carcinoma tissues were pathologically classified according to the World Health Organization criteria [36]. The American Thyroid Association guidelines [11] were followed for therapeutic planning of the patients. Depending on clinical indication, noninvasive examinations included chest radiography, CT, MRI, bone scintigraphy, PET-CT, and ^131^I-scintigraphy may be performed. After thyroidectomy, thyroid remnant ablation was performed between four and six weeks after surgery for the patients with intermediate or high risk. The ^131^I ablation dose for most of the patients was 1.1-3.7 GBq (30-100 mCi). WBS was performed one week after ^131^I administration using the dual-head gamma camera (model of Dual head Genesys, Philips/ADAC, Stokesdale, NC; and model of Infinia Hawkeye 4, GE Healthcare, Haifa, Israel) equipped with a high-energy collimator. Subsequently, treatment with L-T_4_ was initiated in order to decrease the level of TSH without inducing clinical thyrotoxicosis. Cases in which the foci of ^131^I uptake extended beyond the thyroid bed were classified as distant metastasis. This type of patients received higher therapeutic doses of 3.7-7.4 GBq (100-200 mCi). Hospital isolation was arranged and ^131^I-WBS was performed two weeks after administration of ^131^I.

### Enrichment and isolation of CECs

A negative selection system PowerMag was used to enrich CECs [37]. Briefly, fresh blood samples from patients or healthy donors were processed by lysis of red blood cells followed by depletion of CD45^+^ white blood cells using a magnetic chamber. The viable CECs were enriched from the patients and analyzed subsequently as described previously [38, 38].

### Immunofluorescence staining and CEC enumeration

For immunofluorescence staining, leukocyte-depleted cell filtrates were separated into two aliquots. One of the aliquots was incubated with the anti-TSHR antibody (Abcam Inc, Cambridge, England) and the DNA staining dye Hoechst 33342 (Invitrogen Inc, Carlsbad, CA) at room temperature for 1 h. The other aliquot was incubated with the anti-EpCAM antibody (Abcam Inc, Cambridge, England) and the DNA staining dye Hoechst 33342 at room temperature for 1 h. After several washes and centrifugation to remove the supernatants, the cell pellets were resuspended and the Alexa Fluor 488-conjugated donkey anti-mouse antibody (Invitrogen Inc, Carlsbad, CA) was added to the cell suspension. After incubation in the dark for 30 min, the unbound antibody was removed and immunofluorescent images were captured and analyzed by fluorescence microscopy (Zeiss Axiovert 200M). EpCAM^+^-CEC was defined as the cell that was positive for Hoechst 33342 and EpCAM. TSHR^+^-CEC was defined as the cell that was positive for Hoechst 33342 and TSHR.

### Statistical analysis

The CEC counts in healthy controls and in the patients with PTC were compared using the Kruskal-Wallis test for all groups. Dunn's test was used for post-hoc test between any two groups. ROC analysis was used to illustrate the discrimination ability between any two groups. The follow-up year and the cumulative RAI dose for G2 and G3 were compared and analyzed by using the Mann-Whitney test. Statistical analysis was performed using SPSS for Windows (version 18, SPSS, Chicago, IL). A *p*-value < 0.05 was considered statistically significant.

## SUPPLEMENTARY MATERIAL TABLES



## Abbreviations

Anti-TgAb: anti-thyroglobulin antibody
AUC: area under the curve
CEC: circulating epithelial cell
CT: computed tomography
CTC: circulating tumor cell
EpCAM: epithelial cell adhesion molecule
FVPTC: follicular variant of PTC
MRI: magnetic resonance imaging
PET: positron emission tomography
PTC: papillary thyroid carcinoma
ROC: receiver operating characteristic
T4: thyroxine
Tg: thyroglobulin
TNM: tumor-node-metastasis
TPO: thyroid peroxidase
TSH: thyroid-stimulating hormone
TSHR: thyroid-stimulating hormone receptor
WBS: whole body scintigraphy
