# Supplementary material for: Combined analysis of circulating epithelial cells and serum thyroglobulin for distinguishing disease status of the patients with papillary thyroid carcinoma
Source: Oncotarget. 2015 Dec 13;7(13):17242–53. doi: 10.18632/oncotarget.6587 (PMC4941384; doi:10.18632/oncotarget.6587)
Supplement: Supplementary file 1 [file oncotarget-07-17242-s001.pdf]

Combined analysis of circulating epithelial cells and serum thyroglobulin for distinguishing disease status of the patients with papillary thyroid carcinoma

Supplementary Material

Supplementary Table 1. ROC analysis of EpCAM$^+$-CEC counts in distinguishing G3 from G2.

EpCAM$^+$-CEC (cell/ml)	Sensitivity (%)	Specificity (%)
6	100.0	19.2
7	95.5	26.9
8	95.5	30.8
9	95.5	34.6
10	95.5	46.2
11	90.9	46.2
12	90.9	50.0
13	90.9	53.9
14	90.9	61.5
15	90.9	69.2
16	90.9	73.1
17	90.9	76.9
18	90.9	80.8
20	90.9	84.6
22	86.4	88.5
23	86.4	92.3
24	81.8	92.3
28	81.8	96.2
30	77.3	96.2
36	72.7	96.2
45	68.2	96.2
51	68.2	100.0

Supplementary Table 2. ROC analysis of EpCAM$^+$-CEC counts in distinguishing G3 from G1.

EpCAM$^+$-CEC (cell/ml)	Sensitivity (%)	Specificity (%)
6	100.0	52.9
8	95.5	52.9
9	95.5	58.8
10	95.5	64.7
12	90.9	82.4
14	90.9	88.2
15	90.9	94.1
19	90.9	100.0

Supplementary Table 3. ROC analysis of $TSHR^+$-CEC count in distinguishing G3 from G2.

$TSHR^+$-CEC (cell/ml)	Sensitivity (%)	Specificity (%)
1	100.0	3.8
3	95.5	3.8
4	95.5	7.7
5	95.5	11.5
6	90.9	11.5
8	90.9	15.4
9	90.9	19.2
10	90.9	26.9
11	90.9	30.8
12	90.9	38.5
13	90.9	42.3
14	86.4	46.2
16	86.4	50.0
17	86.4	53.9
18	86.4	57.7
19	86.4	65.4
20	86.4	69.2
22	81.8	69.2
23	81.8	73.1
26	81.8	80.8
29	81.8	88.5
30	77.3	88.5
31	77.3	92.3
32	72.7	92.3
33	72.7	96.2
36	68.2	96.2
52	68.2	100.0

Supplementary Table 4. ROC analysis of $TSHR^+$-CEC count in distinguishing G3 from G1

$TSHR^+$-CEC (cell/ml)	Sensitivity (%)	Specificity (%)
1	100.0	11.8
3	95.5	23.5
5	95.5	29.4
6	90.9	35.3
7	90.9	41.2
8	90.9	47.1
10	90.9	52.9
12	90.9	70.6
13	90.9	76.5
14	86.4	76.5
16	86.4	82.4
19	86.4	88.2
20	86.4	94.1
21	86.4	100.0
